# Amid the COVID-19 Pandemic, Unethical Behavior in the Name of the Company: The Role of Job Insecurity, Job Embeddedness, and Turnover Intention

**DOI:** 10.3390/ijerph19010247

**Published:** 2021-12-27

**Authors:** Ibrahim A. Elshaer, Alaa M. S. Azazz

**Affiliations:** 1 Management Department, College of Business Administration, King Faisal University, Al-Hassa 31982, Saudi Arabia; 2 Hotel Studies Department, Faculty of Tourism and Hotels, Suez Canal University, Ismailia 41522, Egypt; 3 Department of Tourism and Hospitality, Arts College, King Faisal University, Al-Ahsa 380, Saudi Arabia; 4 Tourism Studies Department, Faculty of Tourism and Hotels, Suez Canal University, Ismailia 41522, Egypt

**Keywords:** job insecurity, unethical organizational behavior, job embeddedness, turnover intention, COVID-19, tourism industry

## Abstract

The worldwide economic crisis initiated by the COVID-19 pandemic certainly altered the perception of regular job insecurity dimensions and brought these to the ultimate level. When employees feel insecure, they may decide to participate in unethical behavior in the name of the company to avoid layoff and become retained employees. This study investigated the relationship between job insecurity and unethical organizational behavior through the mediating role of job embeddedness and turnover intention. A total of 685 employees working in five- and four-star hotels and category A travel agents participated in this study. Data were analyzed using structural equation modeling. Job embeddedness and turnover intention were found to be partially mediated by the impact of job insecurity on unethical organizational behavior. Theoretical and practical implications were identified and discussed.

## 1. Introduction

Until the recent outbreak of the COVID-19 virus, which has spread throughout the globe, organizational life in the twenty-first century had never been more challenging. This pandemic, which spread across numerous countries at the same time, has harmed billions of people worldwide [1]. As a response to this pandemic, more than 30 million employees are expected to lose their jobs in the United States [2]. The leisure and hospitality industry was the most impacted, with 7.7 million jobs lost, or 47% of the total positions worldwide [3]. Prior to the current epidemic, the travel and tourist industry had remarkable resilience, contributing more than 10% of global GDP and a similar percentage of jobs [4]. However, due to the lockdowns and bans on internal and international travel executed by a large number of countries, this sector is currently the most affected [5]. Many countries’ hospitality businesses have begun laying off employees to cope with the massive losses. Marriott International hotel chains^®^ have begun to lay off tens of thousands of employees around the world [6]. Similarly, Airbnb^®^ laid off almost a quarter of its workforce [7]. Even employees who survive the layoffs are apprehensive about their future careers and have a high level of job insecurity under these conditions. According to previous research, job insecurity is one of the most significant hindrance-related stressors [8,9,10] that has negative impacts on the hospitality industry’s desirable work outcomes [11,12]—and causes absenteeism, nervousness [13], and higher turnover intentions [14].

Though job insecurity has been thoroughly researched, further research can explore the specific employee’s reactions to adapt and deal with job insecurity [8,15]. According to [16], job insecurity can be combined with high achievement to eliminate work withdrawal. Additionally, employees with a higher feeling of job embeddedness can use their capabilities to avoid layoff and become retained employees [17]. They may wish to show their managers that they can make a positive contribution to the company, especially in a time when job instability is on the rise [18]. As a result, they may not see any moral boundaries to engaging in unethical behavior actions that are beneficial to the organization [19].

The issue of employees engaging in unethical behavior to contend with the feeling of job insecurity has been previously highlighted in the literature [20]. However, impacted employees could decide to participate in unethical behavior in the name of the organization but contradict the international ethical standards. A salesperson, for example, could exaggerate the characteristics of a product they were selling to a consumer in order to meet their sales target and assist their company to earn more money. Alternatively, an accountant may falsify figures in order to decrease a company’s tax liability [21]. By executing these unethical practices in the name of the company, the employee may be perceived as a successful employee by their managers [21].

The current study first explores the relationship between job insecurity and unethical organizational behavior. This relationship is examined in the context of increasing the fear of job loss with the tendency for employees to engage in unethical behavior that provides short-term advantages to the organization. Second, this study tests the mediating role of job embeddedness in the relationship between job insecurity and unethical organizational behavior, and finally, the study also highlights the mediating effects of turnover intention in the relationship between job insecurity and unethical organizational behavior. Though job insecurity has been extensively investigated, further research can explore the specific employee’s reactions to adapt and deal with job insecurity [8,15]. Thus, this study proposed a model that may contribute to enhancing academics’ understanding in which job insecurity affects employees’ unethical organizational behavior through the mediating role of job embeddedness and turnover intention.

Testing these relationships has implications for practitioners as well. Employees who participated in unethical organizational behavior in the name of the company can be for personal gains and such behaviors can reduce the reputation of the organization. Therefore, managers amid such a pandemic should avoid sending signals that can promote employees’ perceptions of job insecurity.

## 2. Literature Review and Hypotheses Development

### 2.1. Job Insecurity and Unethical Organizational Behavior

Job insecurity is a ‘perceptual phenomenon’ that focuses on the threat to an individual’s current job stability [22]. The authors of [23] propose two dimensions of job insecurity: quantitative ‘threats to the job as such’ and qualitative ‘threats to valued job features’. Quantitative job insecurity emphasizes the predicted job loss caused by intended/unintended organizational signals, or employees’ appraisal reports [22]. Qualitative job insecurity explains an employee’s perceived future job loss based on their presumed threat [22]. Given the substantial negative impact of the COVID-19 pandemic on the economy, which affects both demand and supply, organizational restructuring through downsizing has become a popular approach. Downsizing is a strategy for cutting and controlling labor expenses (typically by reducing the number of employees or lowering compensation), streamlining operations, and boosting organizational competitiveness [24]. According to researchers [25,26], downsizing could threaten employees and their jobs. Organizational restructure, according to [27], increases employees’ job insecurity. Employees may experience employment insecurity as a result of COVID-19. In the same vein, employees often experience stress because of perceived job insecurity. An employees’ stress response frequently stimulates employees to participate in unethical behaviors that help to cope with the perceived threat [28]. Employees can participate in unethical organizational behavior by acting in their self-interest or the best interests of the organization.

Organizational unethical behavior is an unethical practice that is intended to benefit the organization rather than the person [21]. For example, supplying incorrect information to a customer in order to accomplish the organization’s quarterly predetermined goals could be one of these practices.

**Figure 1:**
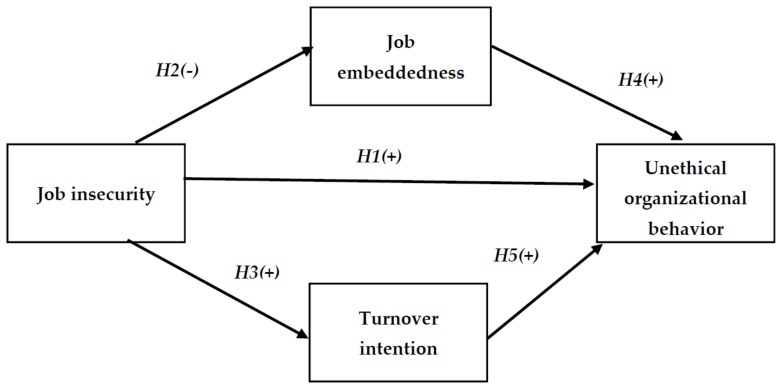
A research framework (developed by authors).

As proposed by the self-regulation theory [29,30], exercising self-control necessitates the application of a limited number of self-regulatory resources. The application of these self-regulatory systems depletes these resources, reducing a person’s ability to demonstrate the self-control required to make ethical decisions. Additionally, when employees’ moral resources have been diminished, their cognitive capabilities are drained, and their successive skill to self-regulate is impeded. When employees’ self-regulatory resources are depleted, employees may decide to participate in unethical behavior that benefits the organization or themselves. Therefore, the following hypothesis is proposed as shown in Figure 1:

**Hypothesis** **1** **(H1).** *Job insecurity has a positive significant impact on unethical organizational behavior.*

### 2.2. Job Insecurity, Job Embeddedness, and Turnover Intention

When the organization members face and feel job insecurity, threats to financial resources can be devastating. The high risk of losing a job threatens the employees’ feelings of embeddedness and fit with the organization. Job embeddedness has been explained as a solid net through which employees at the workplace are attached [31]. The more connections at the workplace that employees have, the more embedded the employee is. The previous literature provides evidence that job insecurity stimulates a lower level of job embeddedness [32].

Employees’ feelings of job insecurity will stimulate a search for new job opportunities and increase the possibilities of turnover. The tourism and hospitality industry is one of the industries with the highest employee turnover rates [33], which may be caused by an unstable environment [34]. The worldwide economic crisis initiated by the COVID-19 pandemic certainly altered the perception of regular job insecurity dimensions and brought these to the ultimate level due to the failure to expect the strength and duration of the pandemic [35]. The meta-analytical research conducted by [36] endorses the claim that job insecurity is a major stressor that is directly related to low job satisfaction and high levels of job withdrawal. Notwithstanding, and based on Adams’ [37,38] equity theory, employees regularly compare their ratio of inputs and outputs as compared to their peers in the organization, and if an imbalance exists an inequity exists. The authors of [36] argue that employees’ feelings of job insecurity could stimulate an imbalanced feeling between their input efforts and output gains. More specifically, employees, compare their organizational loyalty with their perceived job security. On the other hand, as employees’ feelings of job insecurity increase, their intention to leave the organization will increase [39]. Accordingly, the following hypotheses are proposed.

**Hypothesis** **2** **(H2).** *Job insecurity has a negative significant impact on job embeddedness.*

**Hypothesis** **3** **(H3).** *Job insecurity has a positive significant impact on turnover intention.*

### 2.3. Job Embeddedness and Unethical Organizational Behavior

Job embeddedness defines the affective and cognitive connection with the organization, concerned with the organization–employee fit, and builds the internal and external links in the organization and the sacrifices resulting from the breaking of these links [40]. Therefore, it is defined as a set of ‘combined forces’ that bind an employee to the job [41,42]. Job embeddedness refers to a person’s social involvement within their company [43]. As a result, people who are emotionally attached to their jobs are unlikely to leave the company [40].

Embedded workers are aware of the advantages of being attached to their job. Employees with a high level of job embeddedness feel comfortable and compatible with their coworkers, which leads to heightened levels of attachment to the company [44]. While work embeddedness provides heightened degrees of commitment to the company, it also creates an inherent amount of dependence on the organization in terms of job insecurity [44]. According to self-regulation theory, employees make a conscious effort to match their practices and behaviors with accepted norms [45,46]. Employees with higher levels of embeddedness will demonstrate behaviors that are aligned with the organization as a result of increasing levels of fit and connection. Individuals with low levels of embeddedness, on the other hand, have not developed a strong attachment or fondness for the organization [40]. While work embeddedness increases degrees of attachment to the organization, it also produces an inherent level of dependence on the organization, which contributes to job insecurity [44]. Self-regulation theory argued that employees exert a conscious effort to associate their behaviors with established standards [45,46]. Employees who have higher levels of embeddedness will exhibit ethical and/or unethical behaviors in the name of the organization [40]. Therefore, the following hypothesis is proposed:

**Hypothesis** **4** **(H4).** *Job embeddedness has a positive significant impact on unethical organizational behavior.*

### 2.4. Turnover Intention and Unethical Organization Behavior

Employees who are facing turnover intention may participate in unethical organizational behaviors in the hopes that their sacrifices will be rewarded with ongoing employment. However, these unethical behaviors can generate succeeding harms to the organization. On the other hand, employees also may choose to participate in unethical behavior such as padding work hours in an attempt to release some of the disappointment they are suffering regarding the possible job loss [45] and, hence, the following hypothesis is proposed:

**Hypothesis** **5** **(H5).** *Turnover intention has a positive and significant impact on unethical organizational behavior.*

## 3. Methodological Approach

### 3.1. Instrument Development and Research Measures

The current study scales were developed based on a survey of existing theoretical items and a review of the literature. This survey yields four factors, each with its own set of items, which have been customized to fit the tourism sector. The operationalization of the study concepts was derided from previous literature. The study scale was developed using a five-point Likert-type scale anchored by ‘1 = strongly disagree and 5 = strongly agree’, as suggested by [47,48]. Similarly, turnover intention (TrnOvr) was measured by three items developed by [34,49,50]. Job insecurity (JobInsc) was measured by six quantitative and qualitative items adopted from [51] (e.g., ‘I am worried that I will have to leave my job before I would like to’). Job embeddedness (JobEmb) was operationalized by six items developed by [31] (i.e., ‘I like the authority and responsibility I have at this company’). Finally, from Umphress et al. [21], seven items to measure unethical organizational behavior were employed (i.e., ‘If my organization needed me to, I would give a good recommendation on the behalf of an incompetent employee in the hope that the person will become another organization’s problem instead of my own’).

The instrument was created in English at first. Back-translation was then conducted [52]. The research instrument was translated from English to Arabic by three academics. In addition, the back translation from Arabic to English was done by a group of two more distinct academics. Both versions were identical. There were no discernible discrepancies between the original and translated instruments. Five academics in the tourism industry, thirty employees, eleven experts, and managers from twenty different hotels were used to validate the research instrument. The pilot respondents provided positive feedback on the consistency, content, and face validity of the scale. The final form of the scale was directed to 700 employees working in five- and four-star hotels in Egypt.

### 3.2. Data Collections

The drop and collect method of distributing and collecting the study questionnaires was employed to ensure a high response rate [53]. Survived employees, who may have an intention to leave the hotel amid the COVID-19 pandemic, were targeted to answer the study instrument. Twenty-five enumerators (faculty students) were instructed to collect data from the respondents in greater Cairo, Hurghada, and Sharm Elsheikh (the biggest tourist cities in Egypt). This method was employed to avert the usual low response rate of mail and/or online approach of data collection and to avert the reluctance to answer the anonymous questionnaires. Enumerators were taught to follow hygiene protocols to minimize the risk of infection for themselves and respondents amid the data collection process during July and August 2021. Respondents were asked to sign a consent form before starting the survey.

With a usable response rate of 97%, 685 employees working in the Egyptian five- and four-star hotels and travel agent category A participated in the study survey. A total of 65 four-star hotels, 60 five-star hotels, and 60 category A travel agents were represented in the survey. Four/five questionnaires were sent to each hotel/ travel agent to deal with over-or under-representation. The majority (51%) of the respondents were aged between 31 to 40 and were married (66%). The distribution of the respondents according to gender is nearly equivalent, with 55% male and 45% female. The majority of respondents were normal employees (85%), while only 15% were supervisors. The full-time employees comprised the highest percentage, at 86%, as did employees who had obtained a university degree (85%). A high percentage (43%) of respondents have an annual salary below 4000$, as shown in Table 1.

### 3.3. Non-Response and Common Bias Tests

Two different methods were employed to deal with the potential non-response bias: univariate analysis ‘independent samples *t*-test, analysis of the variance (ANOVA) and multivariate analysis ‘multivariate analysis of the variance (MANOVA). The findings of the two tests did not statistically generate any significant discrepancies at a 95% confidence level for early and late respondents [54]. To test the potential common method variance, Harmon’s one-factor test method, as suggested by [55], was conducted. The one factor extracted solution accounts for 25% of the variance, which gives evidence that no one factor accounted for the majority of the variance, implying that common method variance is not fully responsible for our findings.

Questionnaire items had a maximum and minimum value of 5 and 1, respectively. The mean scores for all answers ranged from 3.31 to 4.08, with standard deviation values ranging from 1.230 to 0.603 (see Table 2), indicating that the study data is more dispersed and less condensed around the mean value [56]. Furthermore, the skewness and kurtosis values in Table 2 indicated that the data did not violate the normality rules [57].

## 4. Results

### 4.1. Confirmatory Factor Analysis

Confirmatory factor analysis (CFA) was employed to evaluate the overall model fit with the data and to determine the unidimensionality of the study constructs. Several researchers recommended that (χ2/df) should be less than 3 and that all fit indices, such as Comparative Fit Index (CFI) and Tucker-Lewis Index (TLI), should be greater than 0.9, while root-mean-square error of approximation (RMSEA) and root-mean-square residual (RMR) should be less than 0.08 [57,58,59]. To assess the factors’ reliability and validity, Analysis of Moment Structures (AMOS) v25 was employed to test a first-order confirmatory factor analysis with all of the study’s dependent and independent variables. The result of our CFA model in Table 3 revealed that the overall fit statistics indicate a satisfactory model fit, as all obtained fit statistics met the recommended cut-off values.

Convergent and discriminant validity for each construct were evaluated to determine the construct validity. Table 3 showed that factor loadings for all study constructs’ items are all significant at the 0.001 level, exceeding the minimum criteria of 0.5. Furthermore, all of the research constructs had AVEs greater than 0.5, and the construct reliability values for all four constructs exceed the 0.70 criterion. Overall, the previous results showed good convergent validity, as recommended by [60,61].

Cronbach alpha values, correlation matrix, and the square root of AVEs were utilized to test the discriminant validity [62]. Table 3 shows the average variance extracted (AVE), correlation matrix, and composite Cronbach alphas for the research variables. As shown in Table 3, the square root of AVEs was higher than the off-diagonal values, which represent the correlations among those constructs, confirming discriminant validity for research factors as suggested by [62]. Moreover, the average variance extracted (AVE) scores for job insecurity (0.797), job embeddedness (0.899), turnover intention (0.789), and unethical organizational behavior (0.859) exceeded the maximum shared variance (MSV) (ranging from 0.021 to 003), further confirmed that the discriminant validity is supported, as suggested by [59,62]. Additionally, for discriminant validity, the inter-correlations scores for each factor (below diagonal value) should not surpass the square root values of the AVE for each factor (bold diagonal) as shown in Table 3, which further support the discriminant validity of the research variables.

### 4.2. Structural Equations Modeling (SEM) Results

**Figure 2:**
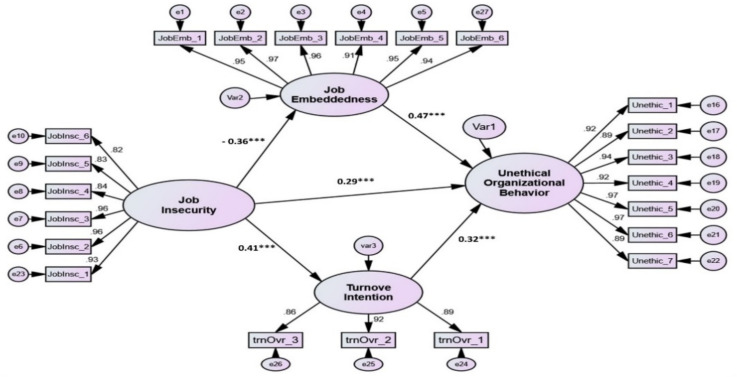
Research model (developed by authors). *** Significant level is less than 0.001.

After ensuring that the validity and reliability of the measures were adequate, structural equation modeling was employed to test the impact of job insecurity on unethical organizational behavior via job embeddedness and turnover intention. Two criteria are employed to assess the proposed model: overall goodness of model fit “χ2/df, CFI, TLI. RMSEA, and RMR” and the statistical significance of the hypothesized relationships. The overall model fit values for the structural model demonstrated satisfactory values, as displayed in Table 4. Moreover, Figure 2 and Table 4 explain the proposed model output.

The relationships in the proposed model involving the five hypotheses investigate the impact of job insecurity on unethical organizational behavior via job embeddedness and turnover intention. The results show that the four paths (H1, H3, H4, and H5) are positive and significant with *p* < 0.05, whereas one path was negative but significant (H2). The significant positive effect of job insecurity on unethical organizational behavior had been supported (β1 = +0.29 with t-value = 6.320, *p* < 0.001). Nevertheless, job insecurity has a significant but negative link with job embeddedness that supports H2 (β2 = −0.36 with t-value = −9.448, *p* < 0.001). The model findings also demonstrate that job insecurity significantly and positively impacts turnover intention (β3 = +0.41 with t-value = 10.221, *p* < 0.001) that proves H3. As assumed in H4, job embeddedness has a positive and significant effect on unethical organizational behavior (β4 = +0.47 with t-value = 11.116, *p* < 0.001) that endorses H4. Similarly, turnover intention was found to have a positive significant impact on unethical organizational behavior, which supports H5 (β5 = +0.32 with t-value = 7.252, *p* > 0.001).

The power of the tested structural model is further proven by the significant coefficient of determination (R^2^) value of 0.42 percent of the variance in unethical organizational behavior can be explained through job insecurity, job embeddedness, and turnover intention.

Additionally, besides the previous direct relationships, the Amos output can provide further information about the indirect effects that can be employed to test the mediation effects in the tested model. To investigate the mediation of job embeddedness and turnover intention in the relationship between job insecurity and unethical organizational behavior, the recommendations of [63,64] were adopted. According to Zhao et al. [64], for a direct-only non-mediation impact, only a direct relationship must exist and be significant; for complementary mediation, both direct and indirect effects must exist and be significant with the same signs. Finally, if both direct and indirect effects are significant with opposite signs, competitive mediation is obtained.

Accordingly, as pictured in Figure 2 and displayed in Table 4, the direct path from job insecurity to turnover intention is positive and significant (β = +0.29, *p* < 0.001); and turnover intention positively and significantly affects unethical organizational behavior (β = +0.32, *p* > 0.001), hence complementary mediation is supported for the mediation effect of turnover intention in the relationship between job insecurity and unethical organizational behavior. On the other hand, the direct path from job insecurity to job embeddedness is negative but significant (β = −0.36, *p* < 0.001); and job embeddedness positively and significantly affects unethical organizational behavior (β = +0.47, *p* > 0.001); hence, competitive mediation is supported for the mediation effect of job embeddedness in the relationship between job insecurity and unethical organizational behavior.

## 5. Discussion and Contributions

The COVID-19 pandemic has spread across multiple countries at the same time and has harmed numerous industries, including the hospitality industry. Multiple approaches have been used to flatten the COVID-19 curve, including lockdowns, social distancing measures, quarantine at home, and travel restrictions, resulting in the temporary closure of many hospitality organizations [65]. Accordingly, hospitality businesses have reacted to the massive losses experienced amid the pandemic by laying off most of their employees. Because employees in developing countries (i.e., Egypt) may be more exposed to job insecurity as a result of inadequate employment protection laws or poor economic environments [66,67], the current study has an exceptional context by testing the impacts of job insecurity on unethical organization behavior among hospitality employees (hotels and travel agents) in a developing country (i.e., Egypt) amid the COVID-19 pandemic.

This paper has attempted to explore and understand the psychological process through which unethical behaviors and decisions are conducted by employees who encountered job insecurity. A total of 650 employees working in the hotel industry and travel agent companies were surveyed to better explain and predict in what way and under what circumstances employees faced with potential job loss amid the COVID-19 pandemic are prone to participate in unethical behaviors. Job embeddedness and turnover intention were employed as mediating variables.

Consistent with the expectations and previous studies’ results [68,69,70,71,72,73,74], job insecurity was found to reduce job embeddedness, reinforce the turnover intention, and promote employees’ unethical organizational behavior. However, there are scarce studies conducted in non-Western countries on these relationships [75]. Consequently, this research contributed to the literature by studying these relationships in the Egyptian context. Scholars have found that employees overcome the perception of job insecurity by working hard, seeking help from others [34,76], and engaging in impression management [77]. However, there is scarce research that examines employees’ reactions to job insecurity by practicing behaviors that are unethical but are in the name of the company.

Job insecurity was found to directly increase employees’ unethical behavior in the name of the company. Employees may practice unethical organizational behavior that may, in turn, assist them to be perceived as valuable to the organization and, accordingly, retain employment or employment benefits. This result is consistent with [21], in which it was found that job insecurity promotes employees’ unethical behavior.

Following [74,78], job insecurity was found to have a negative impact on employee embeddedness to the organizations, especially amid the COVID-19 pandemic. When employees feel the risk of future job loss and insecurity, they begin to reconsider their job and their future career path in the company [32]. This causes them to lose association with their supervisors and destruction in the match and alignment between their beliefs and values and those of the organization. Due to a lack of empirical study on the factors that influence job embeddedness [79], this result can enhance our understanding of such a relationship. Job embeddedness, in turn, was found to promote employees’ unethical behavior in the name of the company. This result is consistent with [31], who argued that, as a result of increased levels of alignment and attachment between the employees and the company, employees with higher levels of embeddedness will exhibit unethical behaviors in the name of the organization.

Additionally, the current study gives evidence that when employees feel insecure, their turnover intention is increased. This result is consistent with the study by [80]. Furthermore, several previous studies have shown that job insecurity impacts job dissatisfaction and intention to quit the job [81,82]. Turnover intention, in turn, can increase the employees’ unethical behaviors in the name of the company in the hopes that their sacrifices will be rewarded with ongoing employment.

This study provides two contributions to practitioners and academics. First, job insecurity should be a high priority for top-level management and human resource managers in hospitality organizations because it leads to a variety of negative consequences not only for employees but for the organization as well. These consequences can include reduced job embeddedness, low job satisfaction, reduced trust in management, poor organizational performance, increases in unethical organizational behavior, and high turnover intention. The study, as well, highlighted the mediating role of job embeddedness and turnover intention in increasing the effect of job insecurity on unethical organizational behavior, as the direct impact of job insecurity on unethical organizational behavior was further strengthened through these two mediators. Testing these relationships may enhance academic’s understanding of the nature of the relationships between job insecurity and unethical behavior.

Second, the study has further implications for managers in the hospitality industry. In the context of a developing country (e.g., Egypt), where unemployment levels are substantially high [8], job insecurity amid the pandemic may have destructive outcomes for hospitality businesses. Perceived job insecurity may threaten the reputation and goodwill of the hospitality industry due to employee’s practicing unethical behavior in the name of the company to retain employment or employment benefits. Consequently, amid such a severe pandemic, managers in the hospitality industry should avoid sending out signals that may cause their employees to believe that they are in danger of losing their jobs. Any uncertainty or miscommunication on the side of management can lead to workers’ feelings of insecurity, resulting in low job embeddedness and high turnover intention, and can promote unethical behavior in the name of the company.

## 6. Study Limitations and Directions for Future Research

This study has four limitations. First, job embeddedness and turnover intention were found to be partially mediated by the impact of job insecurity on unethical organizational behavior. However, other variables (e.g., justice, job satisfaction, and trust in supervisor,) may also intervene in this relationship. As a result, future studies should look at whether the impacts are direct or are mediated by factors other than job embeddedness and turnover intention. Second, due to the cross-sectional nature of the data obtained, causal correlations among the variables cannot be deduced. Third, although we attempted to avoid common technique bias [36], future researchers could employ longitudinal data or a variety of data sources. Fourth, a different model can be employed to test these relationships in different contexts using a multi-group analysis technique [83].

## Figures and Tables

**Table 1:** The Demographic characteristics (developed by authors).

N = 685		%	Groups		Number of Responses
**Age**			Five-star Hotels	65	235
21–30	185	27	Five-star Hotels	60	210
31–40	350	51	Travel agents	60	240
>41	150	22	Total		685
**Gender**					
Male	380	55			
Female	305	45			
**Marital status**					
Married	450	66			
Unmarried	235 200	34 20			
**Occupation**					
Supervisors	105	15			
Normal employees	580	85			
**Type of employment**					
Full time	590	86			
Part time	95	14			
**Education level**					
Less than high school degree	185 200	27 20			
High school degree	100	15			
University graduate	400	58			
**Annual Salary ($)**					
Under 4000	300	43			
4001–6000	150	22			
6001–8000	150	22			
Over 8000	85	13			

**Table 2:** Descriptive statistics (developed by authors based on previous literature).

Abbr.	Items	Min	Max	M	S.D	Skewness	Kurtosis
**Job Insecurity**							
JobInsc_1	“I am worried that I will have to leave my job before I would like to”.	1	5	3.33	1.086	−0.353	−0.422
JobInsc_2	“I worry about being able to keep my job”.	1	5	3.33	1.101	−0.328	−0.489
JobInsc_3	“I am afraid I may lose my job shortly”.	1	5	3.36	1.060	−0.343	−0.385
JobInsc_4	“I worry about getting less stimulating work tasks in the future”.	1	5	3.31	1.127	−0.399	−0.364
JobInsc_5	“I worry about my future wage development”.	1	5	3.31	1.123	−0.404	−0.344
JobInsc_6	“I feel worried about my career development in the organization”.	1	5	3.31	1.140	−0.432	−0.328
**Job Embeddedness**							
JobEmb_1	“I like the members of my workgroup”.	1	5	3.57	1.220	−0.394	−0.953
JobEmb_2	“My coworkers are similar to me”.	1	5	3.54	1.169	−0.334	−0.918
JobEmb_3	“My job utilizes my skills and talents well”.	1	5	3.59	1.162	−0.328	−0.957
JobEmb_4	“I feel like I am a good match for this company”.	1	5	3.47	1.191	−0.324	−0.938
JobEmb_5	“I fit with the company’s culture”.	1	5	3.51	1.184	−0.346	−0.904
JobEmb_6	“I like the authority and responsibility I have at this company”.	1	5	3.62	1.184	−0.396	−0.953
**Turnover Intention**							
trnOvr_1	“I often think about leaving that career”.	1	5	4.08	.618	−1.439	1.485
trnOvr_2	“It would not take much to make me leave this career”.	1	5	4.07	.629	−1.463	1.198
trnOvr_3	“I will probably be looking for another career soon”.	1	5	4.08	.603	−1.439	1.993
**Unethical Organizational Behavior**							
Unethic_1	“If it would help my organization, I would misrepresent the truth to make my organization look good”.	1	5	3.85	1.203	−1.073	0.275
Unethic_2	“If it would help my organization, I would exaggerate the truth about my “company’s products or services to customers and clients”.”	1	5	3.76	1.230	−0.974	−0.020
Unethic_3	“If it would benefit my organization, I would withhold negative information about my company or its products from customers and clients”.	1	5	3.80	1.208	−1.016	0.171
Unethic_4	“If my organization needed me to, I would give a good recommendation on the behalf of an incompetent employee in the hope that the person will become another organization’s problem instead of my own”.	1	5	3.79	1.224	−1.031	0.140
Unethic_5	“If my organization needed me to, I would withhold issuing a refund to a customer or client accidentally overcharged”.	1	5	3.76	1.222	−0.963	0.031
Unethic_6	“If needed, I would conceal information from the public that could be damaging to my organization”.	1	5	3.75	1.250	−0.981	−0.013
Unethic_7	“I would do whatever it takes to help my organization”.	1	5	3.74	1.245	−0.945	−0.085

**Table 3:** Convergent and discriminant validity (developed by authors).

Dimensions and Items	Loading	CR	AVE	MSV	1	2	3	4
1-Job Insecurity (*a* = 0.965)		0.9590	0.7970	0.0210	0.8930			
JobInsc_1	0.934							
JobInsc_2	0.965							
JobInsc_3	0.962							
JobInsc_4	0.836							
JobInsc_5	0.829							
JobInsc_6	0.818							
-2-Job Embeddedness (*a* = 0.981)		0.9820	0.8990	0.003	−0.055	0.9480		
JobEmb_1	0.948							
JobEmb_2	0.974							
JobEmb_3	0.958							
JobEmb_4	0.913							
JobEmb_5	0.952							
JobEmb_6	0.943							
3-Turnover Intention (*a* = 0.918)		0.9180	0.7890	0.0210	0.1440	0.0230	0.888	
trnOvr_1	0.887							
trnOvr_2	0.916							
trnOvr_3	0.861							
4-Unethical Organizational Behavior (*a* = 0.978)		0.9770	0.8590	0.0130	−0.116	−0.040	0.0370	0.927
Unethic_1	0.916							
Unethic_2	0.885							
Unethic_3	0.936							
Unethic_4	0.916							
Unethic_5	0.975							
Unethic_6	0.968							
Unethic_7	0.886							

Model fit: (χ2 (203, N = 685) = 585.046, *p* < 0.001, normed χ2 = 2.882, RMSEA = 0.049, SRMR = 0.050, CFI = 0.937, TLI = 0.924, NFI = 0.938, PCFI = 0.797 and PNFI = 0.816). CR: composite reliability; AVE: average variance extracted; MSV: maximum shared value; Diagonal values: the square root of AVE for each dimension; Below diagonal values: intercorrelation between dimensions.

**Table 4:** Result of the structural model (developed by authors).

	Hypotheses		Beta (β)	C-R (T-Value)	R^2^	Hypotheses Results
H1	Job Insecurity	Unethical organizational behavior	0.29 ***	6.320		Supported
H2	Job Insecurity	Job embeddedness	−0.36 ***	−9.448		Supported
H3	Job Insecurity	Turnover intention	0.41 ***	10.221		Supported
H4	Job embeddedness	Unethical organizational behavior	0.47 ***	11.116		Supported
H5	Turnover intention	Unethical organizational behavior	0.32 ***	7.252		Supported
Unethical organizational behavior					0.42	

Model fit: (χ^2^ (204, N = 685) = 612.204, *p* < 0.001, normed χ^2^ = 3.001, RMSEA = 0.050, SRMR = 0.051, CFI = 0.924, TLI = 0.927, NFI = 0.929, PCFI = 0.719 and PNFI = 0.706). *** *p* < 0.00, n/s = not significant.

## Data Availability

Data is available upon request from researchers who meet the eligibility criteria. Kindly contact the first author privately through e-mail.
